# Reasons for non-compliance with quality standards at primary healthcare clinics in Ekurhuleni, South Africa

**DOI:** 10.4102/phcfm.v12i1.2179

**Published:** 2020-05-27

**Authors:** Lebuile J. Mogakwe, Hafisa Ally, Nomasonto B.D. Magobe

**Affiliations:** 1 Department of Nursing Science, Faculty of Health Sciences, University of Johannesburg, Johannesburg, South Africa

**Keywords:** Reasons, non-compliance, quality standards, primary healthcare clinics, Ekurhuleni

## Abstract

**Background:**

The South African Minister of Health stated that compliance with quality standards in health services is non-negotiable as it is fundamental in improving South Africa’s current poor health outcomes, restoring patient and staff confidence in the public healthcare system, achieving widespread sustainable development and providing basic quality healthcare in South Africa. Non-compliance with quality standards, as evidenced by increased quality-related queries from the community, prompted the researcher to explore and describe the reasons for such at primary healthcare clinics in Ekurhuleni.

**Aim:**

This study sought to explore and describe the reasons for non-compliance with quality standards at the primary healthcare in Ekurhuleni in order to propose recommendations to facilitate compliance with quality standards.

**Setting:**

The study was conducted at primary healthcare clinics in Ekurhuleni, one of the metropolitan districts, situated in an area east of the Gauteng province.

**Methods:**

A qualitative, exploratory, descriptive and contextual research design was used for this study. Participants were purposefully selected from the population and consisted of individuals who willingly consented to participate. Twelve semi-structured individual interviews were conducted.

**Results:**

The study findings revealed challenges with management practices, for example, non-involvement in decision-making, lack of support and poor internal communication practices. In addition, challenges with human, material and financial resources were stated as reasons for non-compliance with quality standards.

**Conclusion:**

Recommendations to facilitate compliance with quality standards were described, which included implementation of effective management practices and allocation of adequate healthcare resources required to facilitate such compliance.

## Introduction

To achieve widespread positive health outcomes, compliance with quality standards at primary healthcare (PHC) clinics is essential. William Adelbert Foster as cited by Brown et al.^1^ states that the effort to comply with quality standards is not incidental, but rather ‘it is always the result of high intention, sincere effort, intelligent direction and skilful execution; it represents the wise choice of many alternatives’. The South African National Department of Health’s (NDoH) Policy on Quality in Health Care supports Foster’s claim and further asserts that compliance and commitment to maintaining high-quality standards will assist South Africa to achieve the goal of quality healthcare for all of its citizens.^2^ Oosthuizen et al.^3^ report that compliance with accepted quality standards of care is the key to improve healthcare, while non-compliance with quality standards will not only result in costly legal action but also in the silent complications of sub-optimal care, with morbidity and mortality repercussions.

The South African NDoH’s policy paper on the National Health Insurance (NHI) stipulates that compliance with National Core Standards (NCS) is one of four key interventions that need to happen in order to successfully implement the NHI.^4^ The South African NDoH’s NCS for Health Establishments (HEs) asserts that compliance with quality standards will ensure patients are protected from life-threatening situations.^5^ A provision of quality services is considered a priority by the South African government; hence, the implementation of components contained in the definition of an ideal clinic (IC) is reinforced in order to facilitate compliance with quality standards at the PHC clinics as well as the delivery of quality health services to communities.^6^

As a quality standards’ compliance manager in Ekurhuleni for a period of 2 years, the researcher has observed that there is a pattern of non-compliance with quality standards at PHC clinics in Ekurhuleni. The non-compliance pattern is evident in data gathered from audits, such as monthly supervisory visits, quarterly programme in-depth reviews, clinical programme support and the outcomes of NCS audits. Interventions have been implemented to facilitate compliance with quality standards. These include distributing policies, strategies and guidelines, such as the NDoH’s PHC Supervision Manual, the NCS for HEs in South Africa, the South African Policy on Quality in Health Care, and supporting policies and guidelines regarding clinical programmes. In addition to these reference materials, management development training, quality improvement workshops, meetings, and continuous guidance, support and mentoring have been provided. Reports submitted by district PHC clinic supervisors, clinical programme coordinators and managers indicate that support visits are undertaken to these clinics to assist PHC clinics to comply with quality standards.

At the end of these support visits, remediation measures are discussed and recommendations are made to assist managers to facilitate compliance with quality standards within their respective PHC clinics. Despite all interventions mentioned above, the PHC clinics in Ekurhuleni are still non-compliant with quality standards. Therefore, the aim and the objectives of this study were to explore and describe the reasons for non-compliance with quality standards at the PHC clinics in Ekurhuleni. The results or findings will inform recommendations to assist managers to facilitate compliance with the quality standards within their respective PHC clinics.

## Research method and design

This study utilised a qualitative, exploratory, descriptive and contextual design to explore and describe reasons for non-compliance with quality standards at PHC clinics in Ekurhuleni, as well as suggestions regarding what could be done to facilitate compliance with quality standards.

### Population, sampling and sample size

The population of the study comprised the managers of the 82 PHC clinics in Ekurhuleni. A purposive sample of PHC clinic managers in Ekurhuleni with 5 or more years of experience, who had undergone quality standard audits, including NCS, and who were willing to participate in the study, was used in this study. The sample size of 12 managers who participated in this study was determined by data saturation.

### Data collection

After the permission to conduct the study was granted by the University of Johannesburg and the Ekurhuleni Health District, the researcher distributed the detailed letters to PHC clinic managers, inviting them to participate in the study. All those who met the inclusion criteria and were willing to participate in the study indicated their interest by either attaching a signature or writing their personal details on the invitation letters, which they then handed back to the researcher together with their contact details for further communication. Eighteen PHC clinic managers indicated their preparedness to participate in the study and thus were invited for data collection. The data collection method included in-depth, semi-structured, individual interviews, observations and field notes. The in-depth, semi-structured, individual interviews were conducted until data saturation was reached at the 12th participant, whereby repetitive themes emerged and no new information was forthcoming from the participants. The two common questions asked to all were as follows: ‘what are the reasons for non-compliance with quality standards in this clinic?’ and ‘What can be done to facilitate compliance with quality standards in this clinic?’

The date and time of interviews were fixed (lasting for 30–45 min per individual interview). However, the interviewer had to be flexible to ensure that participants were given sufficient time to share their reasons for non-compliance with quality standards. An experienced interviewer who is well versed in qualitative research conducted the in-depth, semi-structured, individual interviews. As a quality standards compliance officer in Ekurhuleni, the researcher did not participate in the data collection as this might have resulted in bias and the Hawthorne effect. The participants granted permission for the interviews to be audiotaped, and the interviews were transcribed verbatim.

### Data analysis

The data obtained during the in-depth, semi-structured, individual interviews with the PHC clinic managers were analysed using Tesch’s protocol of qualitative data analysis, as described by Creswell.^7^ These steps entailed organising and preparing data for analysis, and reading and rereading the data to obtain a general sense of the information and its overall meaning, and then coding the data according to themes and sub-themes. The researcher and the independent coder held a consensus discussion meeting after the themes and sub-themes were identified to decide on the identified themes and sub-themes.

### Trustworthiness

Trustworthiness was maintained by using the five criteria of Lincoln and Guba,^8^ including credibility, dependability, confirmability, transferability and authenticity. Credibility was achieved during the interviewer’s prolonged engagement with the PHC clinic managers to gain an in-depth understanding of their reasons for non-compliance with quality standards. The researcher described the research process used in this study in such a way that the same study could be replicated, and the audiotapes, transcripts and field notes were kept to enable an enquiry audit. These measures ensured dependability of the data. In this study, a confirmability audit, triangulation of literature sources and a consensus discussion between the researcher and the independent coder ensured confirmability of the findings. Transferability of this study’s findings to other contexts was ensured by providing a thick description of the context in which the study was conducted, the participants and the research method applied. Authenticity was achieved through the audiotaped interviews and the verbatim transcription of collected data.

### Ethical consideration

The following ethical principles were adhered to throughout the study, with the aim of protecting the participants: respect for persons, beneficence and justice in accordance with Dhai et al.^9^ The Faculty of Health Sciences Academic Ethics Committee (REC-01-151-2015) of the University of Johannesburg and the Ekurhuleni Health District Research Ethics Committee (15/05/2015-3) granted ethical approval prior to conducting the study. Before commencement of the semi-structured individual interviews, the participants were again reminded about the purpose of the study and the methodology to be used, including the audiotaping of the interviews. Voluntary written informed consent was then sought from the participants. The information on the consent form indicated that the participants had the rights to withdraw from the study at any time without any consequences. Also, the participants were duly informed that there were no envisaged risks associated with taking part in the study. Rather, the study sought to explore and describe the reasons for non-compliance with quality in order to propose recommendations to facilitate compliance with these quality standards at PHC clinics in the Ekurhuleni. The semi-structured individual interviews commenced only after the participants had read the contents of the consent form and understood them and signed it.

## Findings and discussion

Two main themes and six sub-themes emerged from the data analysis as described in Table 1. The research findings are supported by relevant national and international literature to add to the richness of data and to add meaning to the collected raw data such as Creswell.^7^

**TABLE 1:** Challenges facing managers at primary healthcare clinics in Ekurhuleni, which they viewed as reasons for non-compliance with quality standards.

Main-theme	Sub-themes
1. Management practice challenges as reasons for non-compliance with quality standards at PHC clinics in Ekurhuleni	1.1. Senior management’s failure to include PHC clinic managers in decision-making 1.2. Lack of support from senior management 1.3. Poor internal communication practices.
2. Required resource challenges as reasons for non-compliance with quality standards at PHC clinics in Ekurhuleni	2.1. Human resource constraints 2.2. Material resource constraints 2.3. Financial resource constraints.

PHC, primary healthcare.

### Management practice challenges cited as reasons for non-compliance with quality standards at primary healthcare clinics in Ekurhuleni

Participants expressed challenges with management practices, namely, senior management’s failure to involve PHC clinic managers in decision-making, a lack of support from senior management and poor internal communication practices.

#### Senior management’s failure to include primary healthcare clinic managers in decision-making

Senior management in Ekurhuleni consists of the sub-district managers, health programme managers and district managers. The role of these managers is to oversee and supervise service delivery at HEs (clinics and community health centres) that render PHC service packages. Furthermore, they are expected to provide all types of support, that is, resources, training and empowerment, that are required by the HEs’ managers in rendering PHC services.

Participants stated that they were not involved in decision-making regarding the day-to-day operations (organisation and control) of the PHC clinics, as indicated in the following statements:

‘No, we [*are*] not involved in the decision-making, even though we are running the clinics, we are the Chief Executive Officers (CEOs) of the clinics.’ (P9)

‘We [*are*] not involved in decision-making. We just attend the meetings, which are all about what the principals are saying and everything that they have already decided.’ (P9)

Nooritajer and Mahfozpour^10^ report that participation in decision-making is often neglected in the nursing profession, it is restricted to chief executives and it is disregarded in the area of healthcare. Similar to the notion above, Mosadeghrad states that almost all decisions regarding structures, general goals, policies and even resource allocation are made by the public health sector at the central level, and that managers in public healthcare facilities do not have the autonomy to make and implement strategic decisions.^11^

#### Lack of support from senior management

The study participants stated that one of the reasons for non-compliance with quality standards is senior management’s lack of support as they do not understand and/or attempt to resolve the challenges that participants face. This is evident in the following statements:

‘The other reason for non-compliance is a lack of support from the supervisor; they do not help us resolve challenges we have with regard to reaching our goal of complying with quality standards.’ (P3)

‘There is no support from management because my immediate supervisor is never there for us to discuss our challenges and understand why we are not complying with quality standards.’ (P2)

In his study, Mosadeghrad found that a non-supportive management team affects compliance with quality standards.^11^ Chipeta^12^ corroborated Mosadgherad’s findings by reporting that inadequate support by management challenges staff members’ morale and their compliance with quality standards in healthcare services. Furthermore, healthcare clinic managers stated that they had no one to turn to when they experienced challenges reaching their quality standards’ compliance goals and they needed support to address these challenges.^12^ Munyewende et al.^13^ assert that nursing managers are strategically placed to lead the implementation of many proposed healthcare reforms, such as complying with quality standards, including the NCS. However, nursing managers face several challenges, including sub-optimal supervision and a lack of support from their superiors.

Other participants cited senior management’s physical absence at PHC clinics as a lack of support, and thus as one of the reasons for non-compliance with quality standards.

Participants said:

‘Whenever there is an audit visit for NCS, the manager does not even come to the clinic to support.’ (P5)

‘Management does not come to the clinic to support our quality improvement initiatives to ensure that we are doing the correct thing, and if everything is left to the facility manager to see to finish, it becomes a burden.’ (P7)

‘There is lack of support from management whereby they do not come to the clinics to support us, but only come to blame us for non-compliance with the quality standards, even though we try and explain the reasons why we are not complying.’ (P5)

Nursing managers experience a general lack of support from their supervisors who are physically absent and therefore unable to support them, and yet only interfere or blame nursing managers for wrongdoings. This creates a non-positive practice environment that is not conducive to compliance with quality standards.^13^

Nkosi et al.’s^14^ study reveals that although PHC clinics’ area managers are appointed to supervise and support their clinics, they do not visit the clinics as frequently as recommended. This undermines clinical supervision as a quality improvement initiative, whereby in-depth clinical programme reviews are carried out to ascertain whether or not managers are implementing standards correctly, and to support them with necessary interventions if they are not being implemented satisfactorily.^14^

#### Poor internal communication practices

Participants expressed that they had insufficient or limited time during which to conduct staff meetings and feedback sessions, and cited this as a reason for their non-compliance with quality standards, as stated in the following comments:

‘As a manager I attend the quality assurance workshops for standard operating procedures (SOPs), and when I return to the facility there is no time to give feedback to the staff.’ (P2)

‘It is possible for me to attend meetings without giving feedback to staff members due to the fact that I cannot in any given time ask staff to leave patients in a queue and let them wait while we [*have*] a feedback session or a meeting.’ (P9)

‘We are supposed to have monthly meetings and discuss relevant issues, but because of the time constraints due to the high workload, we do not have such meetings.’ (P7)

Oandasan et al.^15^ reveal that time constraints related to patient workload pressure affect staff members’ inter-professional communication and collaboration, that is, the staff members do not have time to hold meetings to discuss patient care or administrative issues that are relevant to the quality of patient care. This has a detrimental effect on the quality of patient care. Roth and Markova also identified the lack of time to exchange ideas as amongst the reasons directly responsible for ineffective processes.^16^ The PHC facility managers often feel that they do not have sufficient time to conduct meetings with their staff in which to identify and address work problems together and share communications from higher managerial levels.^17^

### Required resource challenges as reasons for non-compliance with quality standards at primary healthcare clinics in Ekurhuleni

The human, material and financial resource constraints were named as reasons for non-compliance with quality standards at PHC clinics in Ekurhuleni, which are described in detail here.

#### Human resource constraints

The human resource constraints, which participants attributed as reasons for non-compliance with quality standards, were the shortage of health workers and its effects, as well as health workers’ poor knowledge and skills.

**Shortage of health workers and its effects:** Managers ascribed the shortage of health workers and its effects as being amongst the reasons for non-compliance with quality standards at PHC clinics. This arises out of the number of healthcare professionals and the number of available consultation rooms, as well as the long time taken to fill vacant positions within the PHC clinics. For example, a level 1 PHC clinic with nine consultation rooms had only five professional nurses. The negative effects of this situation were experienced particularly when one or two of the staff members went on leave or attended training sessions.

The shortage of staff at PHC clinics in Ekurhuleni reportedly affected patients’ waiting times, patients’ workload, cleanliness, drug supply management and pharmacy practices, staff mentoring, clinical supervision and in-service education.

The shortage of nurses results in a high nurse patient workload above the norm of 1:40 and increases patients’ waiting times (exceeding the benchmark of 2 h), which was also cited as one of the reasons for non-compliance with quality standards. Some participants stated:

‘There is always a shortage of nurses, and this results in [*a*] high workload, hence non-compliance with the quality standards (repeatedly, sounding discouraged).’ (P8)

‘Due to the staff shortages, you find that the nurses attend to more patients than they [*are*] supposed to (a target of forty to forty-five). For example, the nurse–patient ratio at my clinic is 1:62.’ (P4)

Primary healthcare clinics with a high patient to nurse ratio may be forced to ‘cut corners’ and spend less time on each patient in order to cope with the high workload, thus jeopardising the quality of care.^10^

One participant stated:

‘[*The*] shortage of nursing staff increases patient waiting times. This affects the quality of care.’ (P1)

Mathibe et al.^18^ state that quality of care is negatively affected by the overwhelming number of patients in relation to the available staff, which results in prolonged waiting times.

A shortage of cleaners and pharmacy personnel affected the PHC clinics’ general cleanliness and medicine management, respectively, and was cited as being amongst the reasons for non-compliance with quality standards. Some participants said:

‘I have shortage of staff, for instance, I have one general worker, and this affects [*the*] cleanliness of the facility, which is one of the key quality standards.’ (P2)

‘There is [*a*] shortage of staff such as pharmacists and pharmacist assistants, and as a result I have to do pharmacy work and my own work; this results in a high number of expired medicines, which I have to account for, whereas I actually do not have time to check on such things.’ (P8)

Corbett and Miller^19^ propose that the insufficient number of cleaners has led to staff members feeling pressurised to work faster because there is often insufficient staff to carry out all the required tasks in one shift. The cleaner shortage and the concomitant increased pressure could demotivate cleaning staff. Oqua et al. propose that insufficient pharmacy personnel are the reason for poor pharmacy practices, which results in poor-quality healthcare services.^20^

The other effects that result from shortages of health workers include the lack of time, which prevents staff from attending in-service training sessions and from performing clinical supervision:

‘There is no time to mentor the new staff because of the staff shortage in relation to the number of services that must be rendered.’ (P3)

‘I do not mentor the staff because I still have to help them and push the patients’ queues because of shortages of staff and the high workloads.’ (P7)

‘Staff cannot be mentored and [there is no time to] see that they implement the quality standards as expected of them due to the high patients’ workload.’ (P11)

Nettleton and Bray report that because of insufficient time and various increased workload commitments, nurse mentoring – as a leading factor for quality improvement – is often denigrated.^21^ Staff shortages and associated high workloads contribute to challenges that nurse mentors experience in the form of limited contact between nurse mentors and nurses and this is supported by Ndwiga et al.,^22^ who reported that this impacted the quality of health services.

Another participant stated:

‘As a manager, I have to see the patients because of a shortage of staff and the high workload, and this leaves me with no time for clinical supervision to identify quality-related issues.’ (P7)

Bradley et al.^23^ stated that because of staff shortages and high workloads within healthcare facilities, the managers find themselves having to assist nurses attend to patients, rather than engaging in clinical supervision. While Hamid et al. reported that because of staff shortages in the public health sector, management cannot afford to send nurses for in-service training.^24^ Although there are plans to increase health workers’ productivity and performance, there are inadequate opportunities for staff to attend in-service education sessions.^25^

**Health workers’ poor knowledge and skills:** Participants expressed the loss of experienced and skilled nurses as a possible reason for non-compliance with quality standards, as evident in the following participants’ quotes:

‘There is loss of experienced nurses that leads to recruitment of new nurses who are inexperienced, and that consequently bring[*s*] deterioration [*of*] standards and litigations may arise.’ (P3)

‘With two or three nurses resigning it’s a problem, because getting one nurse’s skills up to standard … takes lot of time and this affect[*s*] quality care.’ (P2)

Matamane states that the loss of trained, skilled, high-performing and competent nurses is a loss of intellectual capital that leads to a loss of institutional memory and productivity.^26^ Flynn et al.^27^ stated that the aforementioned loss contributes to a decline in the efficiency and effectiveness of healthcare facilities, affects the quality of patient care and can result in serious adverse events, all of which offer the potential for lawsuits. A high employee turnover gives rise to a loss of skills and knowledge and causes many problems for managers, as most newly employed personnel do not have appropriate work experience and still require training.^11^

Other participants ascribed the constraints they face in developing nurses’ knowledge and skills as one reason for non-compliance with quality standards. This is confirmed in the following statements:

‘There is no time for staff to attend workshops that are arranged, hence they are not on board with what is happening.’ (P11)

‘Not all of my staff is trained in NCS, and as a manager I attend [*training*] and have to come back and train my staff.’ (P8)

The World Health Organization^28^ states that the lack of appropriate training is a cause of health workers being unable to provide quality care according to standards; this is attributed to poor training and development, as well as limited opportunities regarding continuing in-service education sessions and workshops to upgrade knowledge and skills. This impedes delivery of quality patient services.^11,29,30^ Flynn et al. assert that the nurses’ inability to attend in-service training and workshops plays a major role in patient negligence and the occurrence of serious adverse events.^27^

#### Material resource constraints

Participants in the study cited material resource challenges, that is, the shortage of medical supplies, stationery, equipment and non-maintenance of available medical equipment and infrastructure, as one possible reason for non-compliance with quality standards. The participants stated that they received neither sufficient healthcare stationery nor health information recording material. This is evident in the following statements:

‘We are expected to manage the antenatal care and antiretroviral (ART) patients, but there is no stationery. I have to leave the clinic and make the copies somewhere else, and this is time-consuming.’ (P9)

‘I struggle because I never receive enough child health records.’ (P8)

‘There are forever stock-outs or shortages of stationery. It seems as if we are working backwards. New health records are initiated every time, but are never sustained, and only to be told that these records are expensive, and therefore one has to forever improvise because there is no continuous supply of such records.’ (P9)

‘We do not have enough health records, especially those printed and supplied by the health department in local government due to the insufficient budget.’ (P11)

Mabelane et al. view the lack or shortage of stationery at PHC clinics as one of the reasons for non-compliance with quality standards and propose that it seriously hampers the provision of quality PHC services.^31^

Another participant said the following with regard to material resources challenge:

‘Due to delays in [*the*] procurement processes we run out of medical supplies and even tell patients to wash and reuse bandages. This compromises quality of care as the patients come back to the clinic with infected wounds.’ (P8)

Shortages of supplies because of delays in payment of suppliers impact the delivery of quality care.^30^ The unavailability of adequate consumables is reported as undermining infection prevention and control measures.^29^

The shortage of medical equipment and failure to maintain medical equipment and infrastructure were expressed as reasons for non-compliance with quality standards at the PHC clinics. This is evident in the following statements:

‘I do not have the necessary equipment to implement and maintain quality standards.’ (P8)

‘You will find equipment that broke in 2009 but [*is*] still not repaired in 2015, and every time when there is an audit on quality standards, the broken equipment [*is*] always found and this reflects badly on our performance as managers.’ (P7)

Awases et al.^32^ reported the unavailability or shortage of working medical equipment as one of the reasons for the non-delivery of desired quality nursing services at public health institutions. Poorly maintained and dysfunctional equipment with their inherent risks reduce health workers’ productivity and this has a profound impact on the quality of care, as it places the patient at risk, particularly when emergency care is needed.^15^ This is what one of the participants said in relation to infrastructural challenges as a reason for non-compliance with quality standards:

‘One does want to comply with standards, but then there are things that are beyond my control, like infrastructural space constraints due to a small clinic, and this makes us fail.’ (P2)

Inadequate infrastructure and the unavailability of space for different programmes in a PHC clinic compromise patient privacy, hamper service delivery and grossly affect the quality of care.^31^

Some participants stated:

‘We have an open-plan [*building*] whereby we are unable to separate the sick babies from the patients with tuberculosis, and this poses a very serious challenge regarding infection control.’ (P2)

‘I have a small clinic, which leads to overcrowding, and due to this one cannot adhere to the correct and infection prevention and control practices; hence, we are found to be non-compliant during the quality standard audits.’ (P12)

The South African Government^33^ states that inappropriate and poorly designed infrastructure is a barrier to infection prevention and control practices. Space constraints at healthcare facilities result in patient overcrowding, which increases the chances of patients contracting nosocomial infections.^34^ Segnon^35^ reports on nurses’ concerns of working in a healthcare establishment with inadequate working space and hazardous environments with poor infection prevention and control practices. This environment is not conducive to performing duties optimally because of poor occupational health and safety standards that compromise nurse and patient safety.^35^

#### Financial resource constraints

The study participants cited financial resource challenges, such as insufficient budgetary allocations, shortage of medical equipment and supplies, and poor maintenance of medical equipment and infrastructure, as affecting the PHC clinics’ operations, resulting in non-compliance with quality standards. The statement regarding monetary allocation is related to the total population of 64 000 served by this particular PHC clinic. The participants considered this allocation to be insufficient in catering for the community’s needs:

‘We are not allocated enough money to operate with, and [*we are*] expected to spend only R10 000 per year on operational issues.’ (P9)

‘The budget that I [*receive*] for my clinic is not enough for my clinic’s average headcount of 12 000 per month.’ (P4)

Wanjau et al.^36^ report that health facilities have to contend with insufficient budgetary allocations that affect the delivery of quality healthcare services in the public health sector.

One participant said:

‘With the very minimum budget that I am allocated, I cannot buy the medical supplies, such as extra blood pressure (BP) cuffs for the vital signs monitor machine that was bought for the clinic with one cuff only.’ (P3)

The under-funding of healthcare facilities results in the non-availability of critical medical supplies, and this affects health workers’ performance and compromises the delivery of high-quality patient care,^37^ as evident in the following quotes:

‘Only R5000 is allocated for stationery per clinic. From the very same limited budget, it is expected that we make copies of the needed health records as a back-up plan and yet still be able to print all the other documents required for service delivery.’ (P9)

‘Due to the insufficient budget, I do not have enough funds to buy cartridges but I have to print and make copies of relevant and important documents for staff.’ (P4)

Mosadeghrad^11^ revealed that inadequate funding results in healthcare facilities being unable to afford an effective, proper information system that requires enough blank patients’ record cards.

One participant said:

‘Due to budgetary constraints we are unable to service our medical equipment. Servicing is expensive and there is no budget for it, this is ridiculous.’ (P10)

Shikabi^38^ reports that healthcare facilities do not have adequate budgets or funding to maintain equipment. Poor health facility maintenance and repair because of low budgetary allocation contributes to the poor structural quality of healthcare services:^39^

‘We are allocated only R4000 per year for the maintenance of the clinic structure, it is not enough.’ (P4)

The South African National Infrastructure Maintenance Strategy^40^ regards a limited budget as a severe constraint to proper infrastructure maintenance, leading to healthcare service interruptions.

## Limitations

This study was conducted with the 12 PHC clinic managers from Ekurhuleni. The study findings are context-bound and cannot therefore be transferred to other settings.

## Recommendations to facilitate compliance with quality standards at primary healthcare clinics in Ekurhuleni

It is evident from the study results that implementation of effective management practices is necessary to ensure that PHC clinic managers are actively involved in the decision-making processes, which will improve their compliance with quality standards. Ekurhuleni’s senior management team needs to implement effective functional support systems and allow time for PHC clinics to conduct meetings and feedback sessions. Lastly, it is recommended that the PHC clinic managers are allocated adequate human, financial and material resources in the healthcare setting to facilitate their compliance with quality standards.

## Recommendations for nursing practice, nursing education and future research

The following recommendations are based on the study findings and can be applied in areas of nursing practice, nursing education and future research:

- *Recommendations for nursing practice*: The existing nursing policies should be reviewed and strengthened to lead, influence and direct compliance with quality standards at PHC clinics. Quality-related aspects, such as a fixed agenda point for nursing management meetings, should be incorporated in order to identify practical and realistic measures that can be implemented to enable compliance with quality standards.
- *Recommendations for nursing education*: The recommendations described above to facilitate compliance with quality standards should be integrated into the curriculum for training nurse managers in order to have trained rising senior nurse managers and to ensure effective implementation of management practices, such as participative decision-making, the employment of effective staff supportive systems and the promotion of internal communication practices.
- *Recommendations for future nursing research*: The perspectives of other health workers such as cleaners, enrolled nursing assistants and others should be sought in terms of how compliance with quality standards at PHC clinics, which is a team effort, can best be facilitated. A similar study should be replicated in another context to obtain different perspectives regarding the facilitation of compliance with quality standards at PHC clinics. A focus group interview that brings PHC clinic managers to a round table discussion would probably generate more unbiased ideas regarding the reasons for non-compliance with quality standards at PHC clinics in Ekurhuleni. Through this opportunity, these managers would collectively raise issues that are not credited to a single individual, thereby carrying more weight for serious consideration.

## Conclusion

This article focused on the reasons for non-compliance with quality standards at PHC clinics in Ekurhuleni. The central theme that emerged from the data analysis process was that PHC clinic managers were faced with challenges that they expressed as reasons of non-compliance with quality standards. Recommendations to facilitate compliance with quality standards were described. The study has made recommendations to nursing practice, policy, nursing education and future research. This will add value to facilitate compliance with quality standards.
